# Supplementary material for: Solid–Liquid Europium Ion Extraction via Phosphonic Acid-Functionalized Polyvinylidene Fluoride Siloxanes
Source: Polymers (Basel). 2020 Aug 28;12(9):1955. doi: 10.3390/polym12091955 (PMC7563938; doi:10.3390/polym12091955)
Supplement: Supplementary file 1 [file polymers-12-01955-s001.pdf]

SUPPORTING INFORMATION

Solid-Liquid Europium ions extraction Via Phosphonic acid-functionalized Polyvinylidene fluoride siloxanes.

Mohammad Wehbi,*[a,b,#] Ahmad Mehdi,[a] Ali Alaaeddine,[b] Nada Jaber[b] and Bruno Ameduri*[,a]

[a]ICGM, Univ. Montpellier, CNRS, ENSCM, Montpellier, France.

[b]Department of Chemistry and Biochemistry, Faculty of Sciences 1, Lebanese University, Rafic Hariri Campus – Hadath, Beirut, Lebanon.

[#]Present address: Laboratoire de Chimie Moléculaire et Thio-organique, École nationale supérieure d'ingénieurs de Caen, 6 Boulevard Maréchal Juin, 14000 Caen, France.

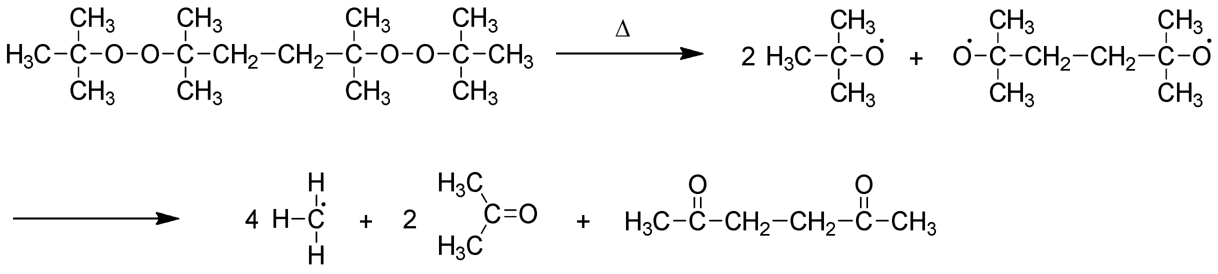

Scheme S1. Mechanism of dissociation of 2,5-dimethyl-2,5-di(*tert*-butylperoxy) hexane (DTBPH) to release various radicals.

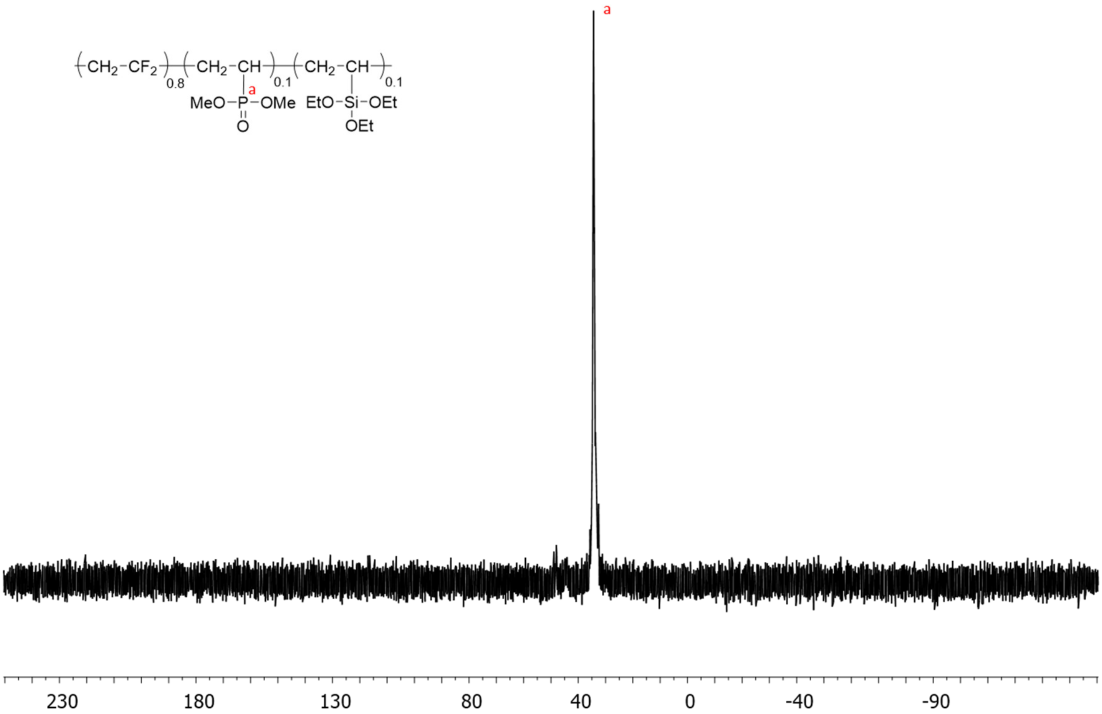

Figure S1. ^{31}P NMR spectrum of poly(VDF-*ter*-VDMP-*ter*-VTEOS) terpolymer (**P_{20}**, Table 1), recorded in DMF-d_7 at 20 °C.

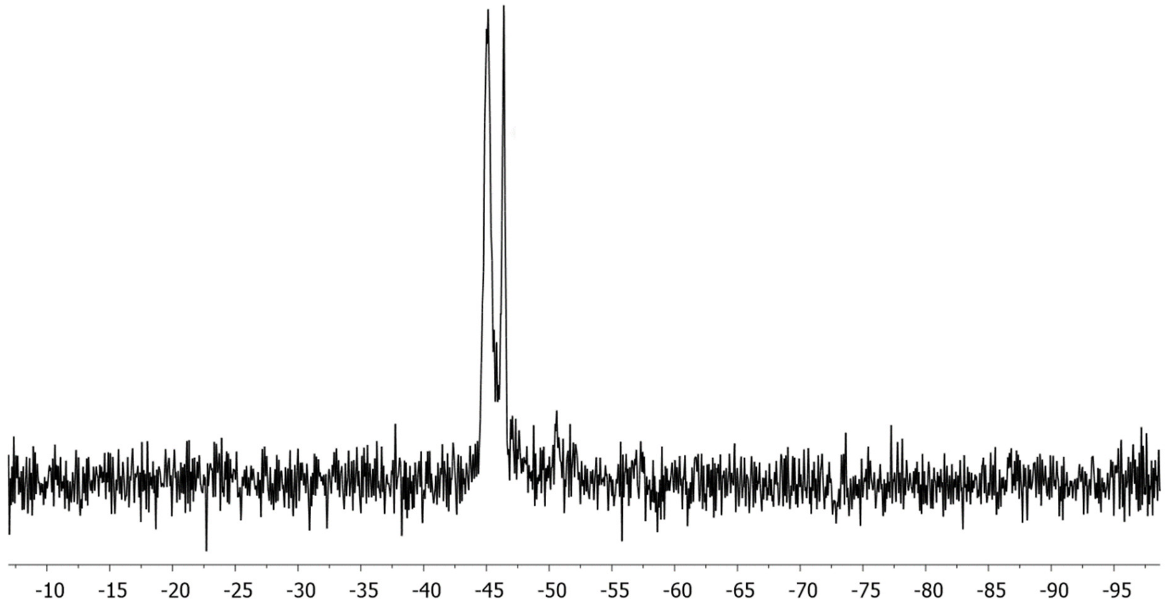

Figure S2. ^{29}Si NMR spectrum of poly(VDF-*ter*-VDMP-*ter*-VTEOS) terpolymer (**P_{20}**, Table 1), recorded in DMF-d_7 at 20 °C.

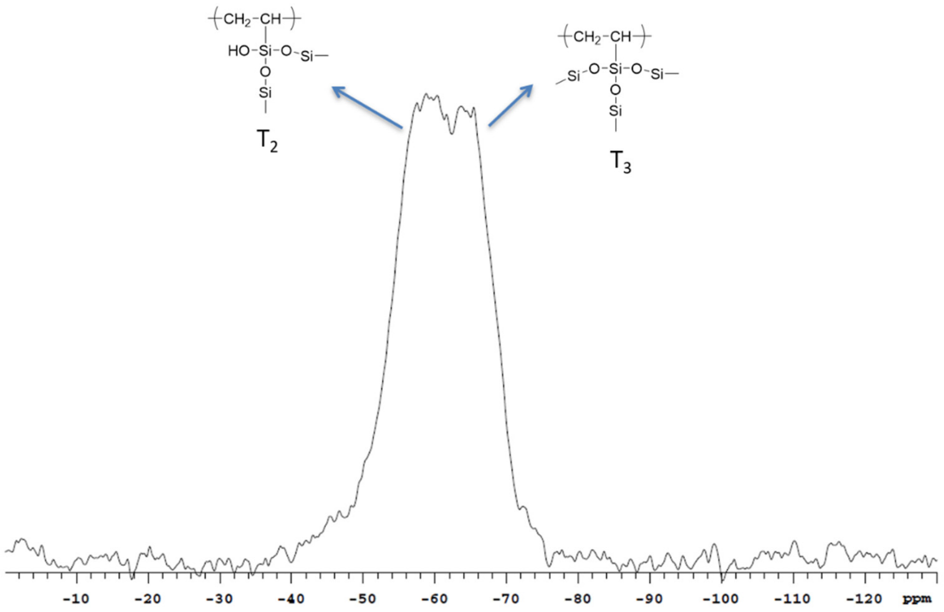

Figure S3. OP-MAS solid state ^{29}Si NMR of poly(VDF-*ter*-VDMP-*ter*-VTEOS) terpolymer after crosslinking (**CP$_{20}$**).

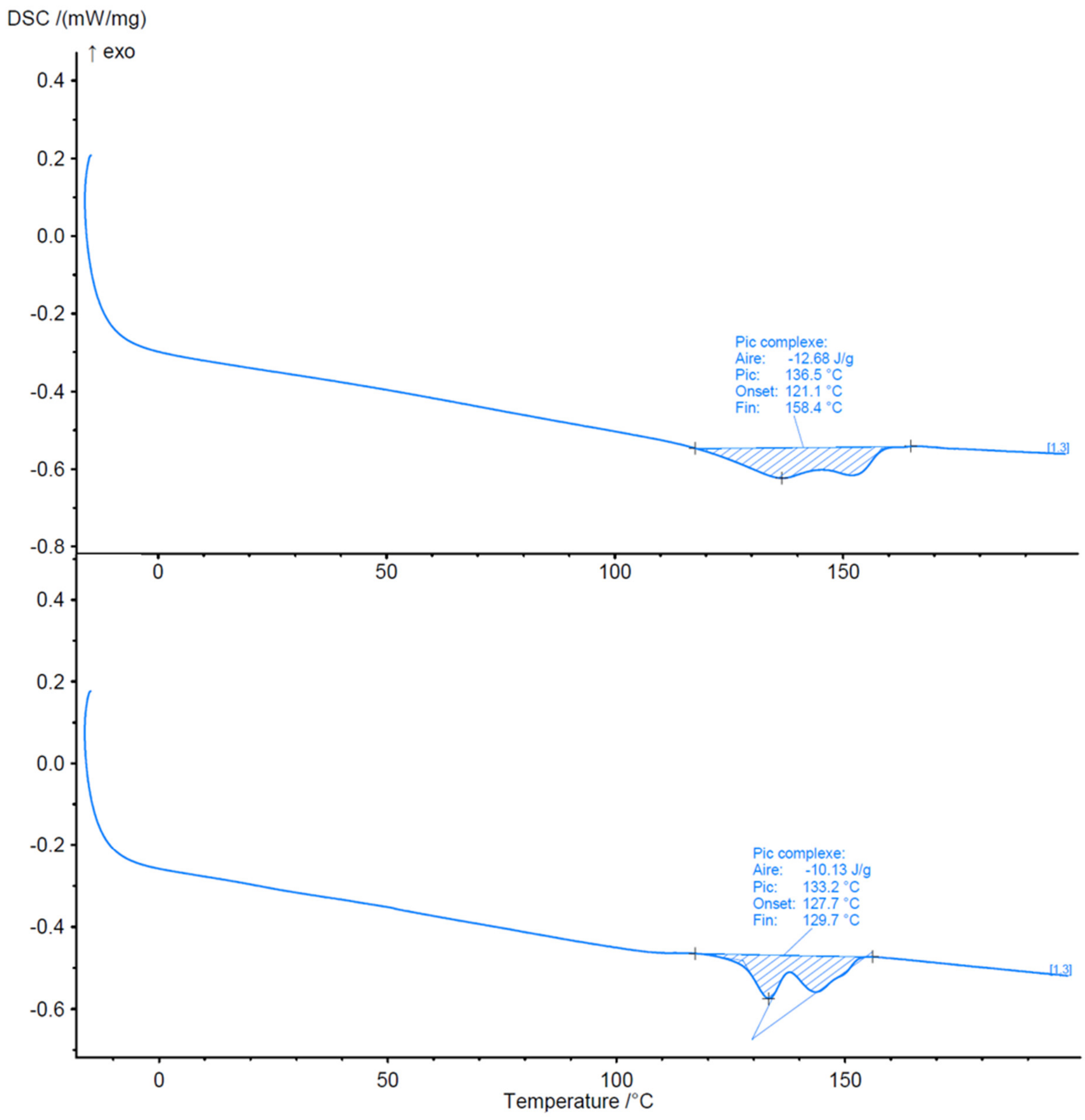

Figure S4. DSC thermograms of poly(VDF-*ter*-VDMP-*ter*-VTEOS) terpolymer (**P**$_{20}$**,** Table 1) before (down) and after crosslinking (top).

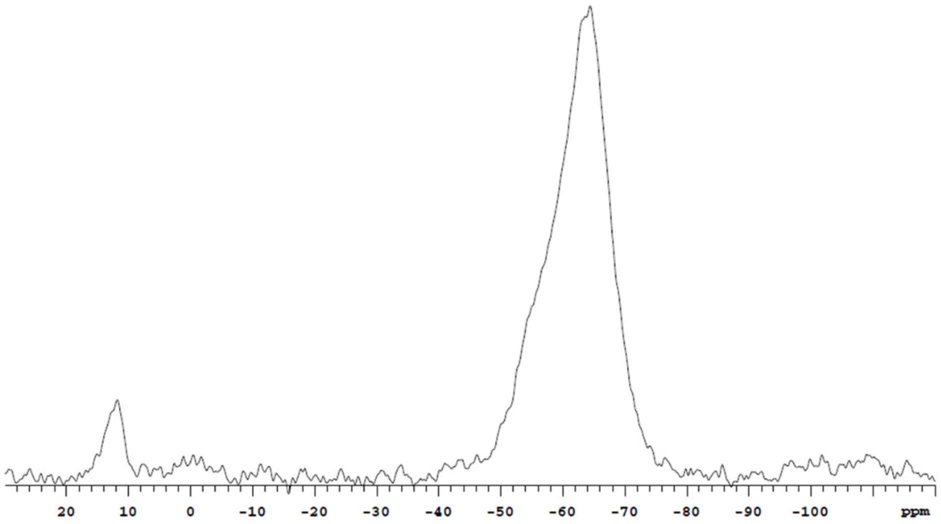

Figure S5. Solid state ^{29}Si NMR spectrum of hydrolyzed crosslinked poly(VDF-*ter*-VPA-*ter*-VTEOS) terpolymer (**HCP$_{20}$**).
